# Fatal Tick-Borne Encephalitis in Unvaccinated Traveler from the United States to Switzerland, 2022

**DOI:** 10.3201/eid3111.251320

**Published:** 2025-11

**Authors:** Chiara Scotti, Gilbert Greub, Yannis Ahmad, Simon Burgermeister, Giovanni Di Liberto, Ekkehard Hewer, Paola Vassallo, Olivier Pantet

**Affiliations:** Lausanne University Hospital and University of Lausanne, Lausanne, Switzerland (C. Scotti, G. Greub, Y. Ahmad, S. Burgermeister, G. Di Liberto, E. Hewer, P. Vassallo, O. Pantet); Queen Square Institute of Neurology, London, UK (P. Vassallo); Leiden University Medical Centre, Leiden, the Netherlands (P. Vassallo); Stichting Epilepsie Instellingen Nederland, Heemstede, the Netherlands (P. Vassallo)

**Keywords:** tick-borne encephalitis, tick-borne encephalitis virus, vaccine, viruses, vector-borne infections, meningitis/encephalitis, flavivirus, Switzerland, United States

## Abstract

We report an unvaccinated traveler from the United States who contracted fulminant fatal tick-borne encephalitis while visiting Switzerland. Climate changes and international travel are intensifying tick exposure for unvaccinated persons. The increasing incidence of tick-borne encephalitis across Europe underscores the importance of tick bite prevention and vaccination against tick-borne encephalitis virus.

Tick-borne encephalitis virus (TBEV), the causative agent of tick-borne encephalitis (TBE), includes 3 subtypes (European, Siberian, and Far Eastern) occurring in Europe and Asia, which are endemic areas. Reservoir hosts are small rodents; transmission occurs mainly by tick bite (*Ixodes ricinus* or *I. persulcatus*) or consuming unpasteurized milk and dairy products from infected animals (*1*). In Central Europe, tick activity is highest in April and May and rises again in September and October (*1*). In Switzerland, ongoing climate change has extended the tick season from March to November, and the area suitable for ticks, which represented ≈16% of the country in 2009, is estimated to >25% (*2*). The number of TBE cases increased from 112 in 2014 to 436 in 2024; the highest incidence was in persons 45–85 years of age (*3*). We report a fatal case of TBE in an unvaccinated traveler from the United States to Switzerland.

On October 13, 2022, a previously healthy 70-year-old US citizen vacationing in Switzerland sought emergency care after 48 hours of experiencing abdominal pain and asthenia. He reported multiple hikes in forested areas in Vaud and Jura cantons in the western part of Switzerland; he had not received TBE vaccination. He experienced rapid neurologic deterioration, urinary retention, ascending paraparesis, and ultimately flaccid tetraplegia. Initial brain and spine magnetic resonance imaging results were unremarkable. Cerebrospinal fluid (CSF) analysis from lumbar puncture revealed increased lymphocytes, hyperproteinorrhachia, and hypoglycorrhachia. On day 5, the patient required orotracheal intubation because of impaired consciousness and bulbar involvement. We diagnosed TBE on the basis of clinical manifestation, typical CSF profile, and positive serum IgM. Testing for TBEV PCR in CSF was negative, as were results of extensive infectious and autoimmune workups. We observed loss of brainstem reflexes and subsequent refractory status epilepticus beginning on day 9. Another MRI revealed the typical TBE pattern (Appendix Figure). We withdrew care on day 16 because of poor prognosis and in accordance with the patient’s advanced directives; the patient died. Autopsy findings (Figure) supported the diagnosis.

**Figure:**
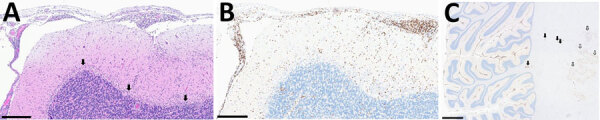
Neuropathologic autopsy findings from cerebellum of unvaccinated traveler from the United States who died of tickborne encephalitis, Switzerland, 2022. A) shows Extensive lymphocytic infiltrate involving both leptomeninges and cerebellar parenchyma with depletion of the Purkinje cell layer (arrows). Scale bar represents 300 μm. B) The infiltrate consisted predominantly of cluster of differentiation 3 + T cell lymphocytes. Scale bar represents 300 μm. C) Low magnification illustrates the diffuse and extensive nature of the infiltrate also involving the white matter with perivascular accentuation (black arrows) and the dentate nucleus (white arrows). Scale bar represents 3 mm. Hematoxylin and eosin staining.

The patient was likely exposed to TBEV during the first part of September 2022 while hiking in a forested area of the canton of Vaud. In that region, estimated TBE incidence in 2024 is 2 cases/100,000 inhabitants, which is lower than in the central and eastern parts of the country (10–23 cases/100,000 inhabitants). The prevalence of TBEV in *I. ricinus* ticks, the main vector of the virus in Europe, may differ between endemic and nonendemic regions of Switzerland, but the overall range is <1% to 14.3% (*4*).

Approximately 75% of TBE infections are asymptomatic (*5*). The median incubation period after a bite is 8 days (*6*–*8*). TBE often follows a biphasic course of a nonspecific febrile illness followed by central nervous system involvement after 4 days (*5*,*9*). Mortality rate can reach 2%; death is associated with older age (>60 years), concurrent conditions, and monophasic illness. Severe neurologic sequelae are reported in ≈10% of patients. EEG is abnormal in 77% of patients with central nervous system involvement (*6*). Diagnosis relies on IgM serology in CSF or serum. IgM is usually detectable at the beginning of the neurologic phase and persists for 3–4 months after infection (*5*). TBE virus RNA can be detected by PCR in the blood during viremic phases and also later in the CSF in ≈75% of patients when neurologic symptoms appear (G. Greub, unpub. data).

TBE treatment relies on supportive care. Vaccination is the most effective preventive measure. Except for the canton of Ticino, risk areas and TBE vaccination recommendations are extended to the entire area of Switzerland for persons >3 years of age (*7*). The World Health Organization and the Advisory Committee on Immunization Practices recommend TBE vaccine for US persons traveling to endemic areas who anticipate substantial tick exposure (*5*). One vaccine is licensed in the United States under the trade name Ticovac (Pfizer, https://www.pfizer.com). For complete primary vaccination, 3 doses should be administered over 1 year. Switzerland’s vaccination plan recommends booster doses every 10 years after the primary vaccination (*3*). Vaccines are safe and effective; estimated protection is >90% after the primary vaccination. A national survey conducted in Switzerland in 2018 showed that TBE vaccination coverage among adults was 41.7% for 1 dose and 32.9% for 3 doses. Data from the children’s vaccination registry for 2022 showed that 50% of children 8–16 years of age had received 3 doses.

During 2000–2023, a total of 12 TBE cases were reported among US travelers in Europe and Asia (*8*). In 2012, the European Centre for Disease Prevention and Control reported 38 cases of internationally acquired TBE (*10*). The incidence is underestimated because of low awareness of TBE endemicity and underreporting of the disease, which is often asymptomatic (*10*). In 2023, a total of 20.8 million tourists visited Switzerland, including 1.4 million from the United States; the increasing tourism from nonendemic countries exacerbates the problem of low awareness of TBEV-related risks. This case emphasizes the importance of personal protective measures and vaccination in travelers to TBE-endemic areas.

Appendix Additional information about a fatal case of tick-borne encephalitis in a traveler from the United States to Switzerland, 2022.
