# Supplementary material for: Fatal Tick-Borne Encephalitis in Unvaccinated Traveler from the United States to Switzerland, 2022
Source: Emerg Infect Dis. 2025 Nov;31(11):2180–1. doi: 10.3201/eid3111.251320 (PMC12704519; doi:10.3201/eid3111.251320)
Supplement: Appendix — Additional information about a fatal case of tick-borne encephalitis in a traveler from the United States to Switzerland, 2022. [file 25-1320-Techapp-s1.pdf]

Article DOI: https://doi.org/10.3201/eid3111.251320

EID cannot ensure accessibility for supplementary materials supplied by authors. Readers who have difficulty accessing supplementary content should contact the authors for assistance.

Fatal Tick-borne Encephalitis in Unvaccinated Traveler from the United States to Switzerland, 2022

Appendix

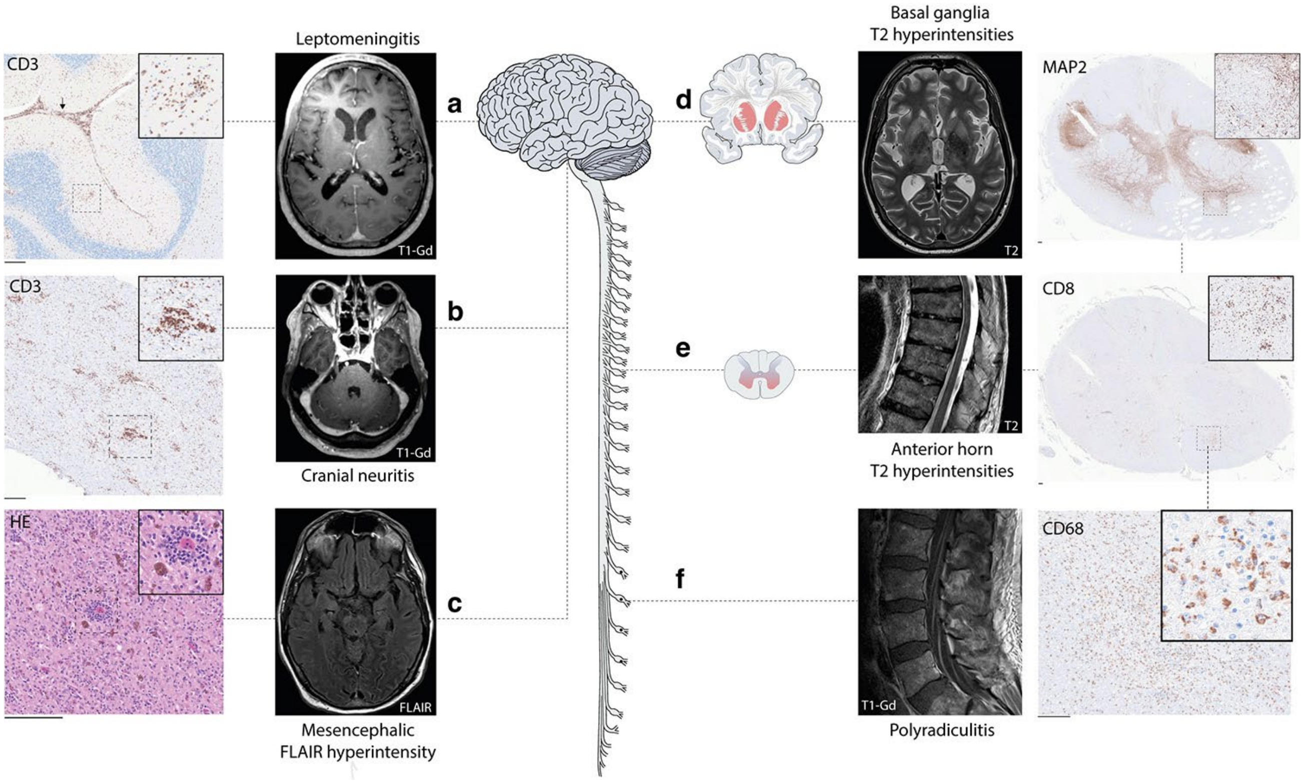

Appendix Figure. Neurologic impact of tick-borne encephalitis (TBE): insights from MRI and histopathological analysis. This figure illustrates the diverse neurologic complications associated with TBE using MRI imaging and histopathological stainings. (a) Leptomeningitis is visualized through T1-weighted MRI with gadolinium, which accentuates meningeal inflammation; accompanying CD3 immunostaining reveals extensive T-cell infiltration of the meninges and cerebellar parenchyma, highlighted by an arrow. (b) Cranial neuritis is illustrated with T1-weighted MRI enhancements of the trigeminal nerve, showing a T-cell predominance in the sheaths of various cranial nerves. (c) Mesencephalic FLAIR hyperintensity is evident from hyperintense FLAIR MRI signals, coupled with hematoxylin and eosin staining that discloses cellular disruptions, including the loss of neuromelanin-

positive cells and lymphocyte infiltration. (d) Basal ganglia exhibit T2 hyperintensities, depicted clearly by T2-weighted MRI. (e) Polyradiculitis is represented by marked contrast enhancement in MRI imaging. (f) T2-weighted MRI of the spinal cord's anterior horn, along with MAP2, CD8 and CD68 immunostaining illustrating extensive loss of motor neurons, infiltration of cytotoxic CD8 T cells and neuronophagia by CD68+ phagocytes. Scale bars: 200 μm for histopathological images.
